# P-2232. Tracheal aspirate and plasma proteomics reveals the local and systemic host immune response to severe pediatric lower respiratory viral infections

**DOI:** 10.1093/ofid/ofae631.2386

**Published:** 2025-01-29

**Authors:** Emily Lydon, Christina M Osborne, Brandie Wagner, J Kirk Harris, Todd Carpenter, Aline Maddux, Matthew Leroue, Jack Kamm, Nadir Yehya, Lilliam Ambroggio, Charles Langelier, Peter Mourani

**Affiliations:** University of California San Francisco Infectious Diseases, San Francisco, CA; Children's Hospital of Philadelphia, Philadelphia, Pennsylvania; University of Colorado, Aurora, Colorado; University of Colorado, Aurora, Colorado; University of Colorado, Aurora, Colorado; University of Colorado, Aurora, Colorado; University of Colorado School of Medicine and Children's Hospital Colorado, Aurora, Colorado; University of California San Francisco, San Francisco, California; Children’s Hospital of Philadelphia, Philadelphia, Pennsylvania; Children's Hospital Colorado, Aurora, CO; University of California San Francisco, San Francisco, California; Arkansas Children's Hospital, Little Rock, Arkansas

## Abstract

**Background:**

Viral lower respiratory tract infections (LRTI) are a leading cause of child mortality worldwide. Improved understanding of local and systemic host immune responses to severe viral LRTI could reveal insights into pathophysiology, lead to novel host-based diagnostic tests, and inform personalized treatment. To date, no studies have yet employed proteomics to simultaneously characterize the lower airway and systemic proteome in pediatric viral LRTI.

Figure 1

**Figure:**
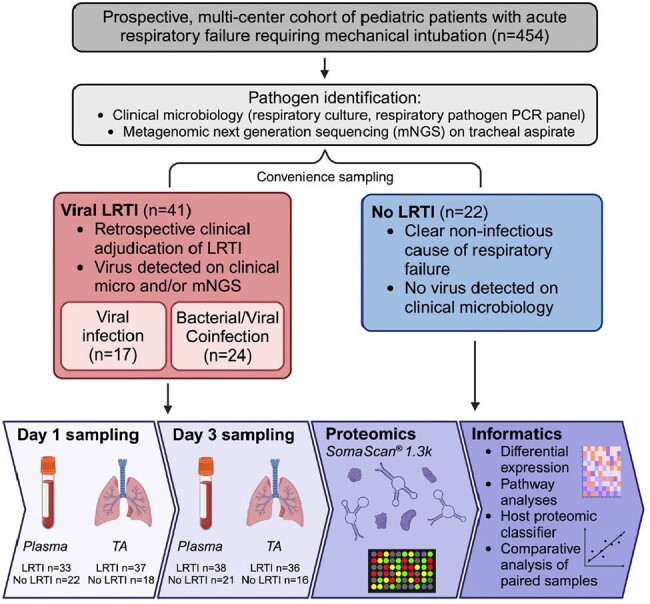

**Methods:**

We used SomaScan^®^ to assess relative expression of 1,305 proteins from tracheal aspirate (TA) and plasma in 63 patients with acute respiratory failure, a subset of a larger prospective multicenter cohort. We performed differential protein expression and pathway enrichment analyses between viral LRTI (n=41; n=24 with bacterial superinfection) and non-infectious respiratory failure (n=22), developed a diagnostic classifier using LASSO regression, and comparatively analyzed TA and plasma samples. Subanalyses were performed to investigate the impact of bacterial superinfection and viral load.

Figure 2

**Figure:**
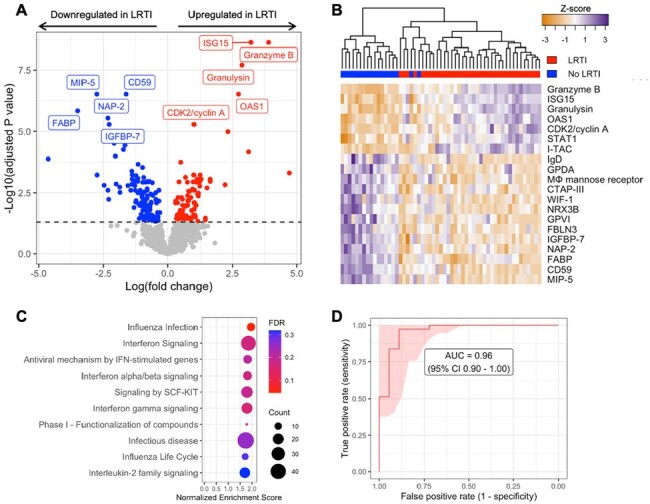

Comparison of host protein expression between Viral LRTI and No LRTI cohorts in tracheal aspirate. (A) Volcano plot of the differentially expressed proteins, with proteins significantly upregulated in LRTI shown in red, and proteins significantly downregulated in LRTI shown in blue. The top ten proteins based on adjusted P-value are labeled. (B) Heat map showing differential expression of the top 20 proteins based on adjusted P-value (rows) across all patients (columns). Dendrogram clustering (top) highlights the proteomic differences between the two groups. (C) Pathway enrichment analysis showing the top ten pathways (all upregulated) ordered by Normalized Enrichment Score. Dot color indicates the false discovery rate (FDR) adjusted P-value, and size indicates the number of proteins included in the pathway. (D) Receiver operator characteristic (ROC) curve of the proteomic classifier to distinguish Viral LRTI from No LRTI.

**Results:**

We identified a distinct proteomic signature of severe viral LRTI in TA with 200 differentially expressed proteins (adjusted P-value< 0.05), with upregulation of proteins key for type I interferon response, NK cells, and cytotoxic T cells and downregulation of inflammation-modulating proteins. A parsimonious diagnostic classifier achieved an AUC of 0.96 (95% CI 0.90-1.00) for diagnosing LRTI. In contrast, the systemic host response to viral LRTI in plasma was more subtle with 56 differentially expressed proteins. Correlation between TA and plasma proteomics was limited, although the interferon-stimulated protein ISG15 demonstrated increased expression across both compartments. Bacterial superinfection showed upregulation of TNF-stimulated protein TSG-6, C-reactive protein, and interferon signaling compared to viral infection alone. Viral load exhibited positive correlation with interferon proteins and negative correlation with neutrophil aggregation proteins.

**Conclusion:**

We characterized the lower airway and systemic proteomic host response of severe pediatric viral LRTI and identified proteins with potential mechanistic role in disease severity that could be targeted with novel interventions.

**Disclosures:**

Jack Kamm, PhD, Genentech: Employee (started there after leaving this study group) Nadir Yehya, MD, AstraZeneca: Advisor/Consultant Lilliam Ambroggio, PhD, Pfizer Inc.: Grant/Research Support

